# Assessing health-related quality of life in patients with interstitial lung diseases

**DOI:** 10.1186/s12890-024-03262-9

**Published:** 2024-09-13

**Authors:** S. Stoltefuß, G. Leuschner, K. Milger, T. Kauke, J. Götschke, T. Veit, A. Lenoir, N. Kneidinger, Jürgen Behr

**Affiliations:** 1 Department of Medicine V, LMU University Hospital, LMU Munich, Member of the German Center for Lung Research (DZL), Marchioninistr. 15, 81377 Munich, Germany; 2 Department of Thoracic Surgery, LMU University Hospital, LMU Munich, Munich, Germany; 3 Division of Pulmonology, Department of Internal Medicine, Medical University of Graz, Graz, Austria

**Keywords:** ILD, Interstitial lung disease, IPF, HRQoL, Health related quality of life, Survey

## Abstract

**Background:**

The R-Scale-PF was proposed to evaluate the health-related quality of life (HRQoL) in patients with idiopathic pulmonary fibrosis (IPF). We generated a German version of the R-Scale-PF (GR-Scale), representing the first translation of the questionnaire into another language and assessed HRQoL longitudinally in various interstitial lung diseases (ILDs) using the R-Scale-PF scoring system at a specialized ILD centre.

**Methods:**

We have translated the questionnaire in accordance with the WHO translation guidelines and applied it to 80 ILD patients of our department, with follow-ups after 3–6 months, assessing its internal consistency, floor and ceiling effects, concurrent validity, known-groups validity, and its responsiveness to changes over time.

**Results:**

At baseline, all 80 patients completed the GR-Scale. In 70 patients (87.5%), follow-up data could be obtained after 4.43 ± 1.2 months. The GR-Scale demonstrated acceptable internal consistency (Cronbach’s α 0.749) and slight floor effects. Concurrent validity analysis showed weak but significant correlations with forced vital capacity (FVC; *r*=-0.282 *p* = 0.011) and diffusion capacity for carbon monoxide (DLco; *r*=-0.254 *p* = 0.025). In the follow-up analysis, moderate correlations were found with FVC (*r*=-0.41 *p* < 0.001) and DLco (*r*=-0.445 *p* < 0.001). No significant difference in the total score was found between patients with IPF (*n* = 10) and with non-IPF ILDs (*n* = 70). The GR-Scale successfully discriminated between groups of varying disease severity based on lung function parameters and the need for long-term oxygen therapy (LTOT). Furthermore, it was able to distinguish between patients showing improvement, stability or decline of lung function parameters.

**Conclusion:**

Our prospective observational pilot study suggests that the GR-Scales is a simple and quick tool to measure HRQoL in patients with ILDs, thus providing an important additional information for the clinical assessment of ILD patients.

**Trial registration:**

Our study was retrospectively registered in the German Clinical Trial Register (DRKS) on 02.11.2022 (DRKS-ID: DRKS00030599).

**Supplementary Information:**

The online version contains supplementary material available at 10.1186/s12890-024-03262-9.

## Introduction

Interstitial lung diseases (ILDs) impose a substantial burden on patients, affecting multiple aspects of their lives, including physical and emotional well-being. Health related quality of life (HRQoL) is often impaired in patients with ILD, especially as the disease progresses and symptoms increase [1–6]. HRQoL is becoming increasingly relevant as a patient-reported outcome and endpoint in clinical trials [7, 8]. Especially in patients with non-curable ILDs, which also impact life expectancy, enhancing or maintaining quality of life is an important therapeutic goal [9, 10]. 

To assess the health status of patients or the HRQoL, patient-reported outcome measures (PROs) can be used, which are typically questionnaires. Hereby, direct information about their health status is obtained from the patients themselves. Information is obtained without the interpretation of an additional person, thus authentically determining the patients’ perspective on their symptom burden and daily life with the disease. Other measures of disease severity and activity, such as pulmonary function tests and chest imaging, which are routinely performed during patient follow-up examinations, undoubtedly provide relevant clinical information. However, in contrast to PROs, these measures only provide information about specific aspects of a disease, but a disease affects the patient’s life and leads to symptoms in various ways. Those measures cannot capture the impact on patients and their lives as a whole, they do not fully reflect how patients “feel, function and survive”. Frequently, there is a discrepancy between those measures and the patients’ perceptions. Therefore, relying exclusively on such parameters can lead to misinterpretations. This highlights the importance of PROs in both clinical trials and in clinical practise [2, 9, 11]. 

Nevertheless, the presence of weak to moderately strong correlations between PROs and follow-up measurements, like lung function parameters, supports the validity of PROs [2, 9].

There are numerous tools to measure HRQoL in patients with ILD. Both disease-unspecific tools like the EuroQol Five-Dimensional Five-Level questionnaire (EQ-5D-5L) or the Short Form 36 Health Survey Questionnaire and disease-specific tools like the King’s Brief Interstitial Lung Disease questionnaire (K-BILD) or the St. George’s Respiratory Questionnaire are currently available. To assess the patients’ HRQoL, the K-BILD questionnaire compromises 15 items and the St. George’s Respiratory Questionnaire 50 items [6, 12–14]. Due to the length and complexity of these questionnaires, they are only very rarely used in clinical practice [6]. Therefore, Scallan et al. proposed the R-Scale-PF (Raghu scale for pulmonary fibrosis) as a new questionnaire to assess the health status in patients with idiopathic pulmonary fibrosis (IPF). The R-Scale-PF is a numerical rating scale, which briefly and visually questions the severity of five symptoms (cough, shortness of breath, fatigue, depressed mood, overall sense of wellbeing) in the last two weeks. The questionnaire has already been employed and evaluated in 100 patients with IPF and did show moderate to high validity compared to established questionnaires like the K-BILD and EQ-5D-5L [6]. 

The R-Scale-PF is currently only available in the original English-language version and has not yet been translated into any other language [6]. A translation of the GR-Scale-PF questionnaire could expand its applicability in clinical practice and research, thereby contributing to its further validation. Translating a PRO like the R-Scale-PF involves a multistep procedure, such as forward-backward translation, in accordance with WHO guidelines [15]. Ensuring the accuracy of the translation is crucial for maintaining the questionnaire’s reliability and the validity. Additionally, it is also essential to ensure the comparability of questionnaire results across different languages [15, 16]. 

The R-Scale-PF was designed to measure HRQoL in patients with IPF and has so far only been used in patients with IPF. Nevertheless, the HRQoL is also limited in patients with other ILD subtypes. In these patients, too, the assessment of HRQoL is becoming increasingly important in clinical studies as well as in everyday clinical practice to capture the impact of the disease on the patients’ lives [3–5, 7]. 

The aim of our prospective observational pilot study was to develop a German version of the R-Scale (GR-Scale) and to evaluate the validity of this GR-Scale in various ILD entities and during follow-up.

## Methods

### Translation of the R-Scale-PF

First, we obtained permission from the copyright holders [6, 17] and translated the questionnaire into German. The translation took place as a forward-backward translation with native English and German speakers and subsequent discussion, according to the WHO translations method [15]. The German version of the questionnaire will be referred to as the “GR-Scale” (German version of the R-Scale). The GR-Scale is shown in the additional file 1. Scores range from 0 to 10 for each individual item and from 0 to 50 for the total score, with higher scores indicating greater limitations.

### Study population and surveys

The study population was recruited at the LMU University Hospital Munich, Germany. We included consecutive individuals with a consensus diagnosis of ILD, including IPF, connective tissue disease-related ILD (CTD-ILD), chronic hypersensitivity pneumonitis (cHP), non-specific interstitial pneumonia (NSIP), sarcoidosis (with present lung parenchymal involvement type III and IV) and unclassifiable interstitial lung disease (uILD). All diagnoses were made in accordance with current international criteria [18]. Patients with an acute infection or other acute illnesses were not included. The patients were interviewed twice, with the follow-up interview taking place after 3 to 6 months. After a short explanation, all patients completed the GR-Scale as self-assessment, without the help of a health care professional. At baseline and follow-up visits, lung function testing (spirometry and gas transfer) was performed as part of the routine assessment.

### Statistical analysis

Descriptive statistics were calculated for baseline variables, using mean and standard deviation to describe parametric data. Frequency tables were created for each item to analyse the floor and ceiling effects, with predefined thresholds at 15% of the participants selecting the minimum or maximum of the items’ scores [19]. Cronbach’s α was assessed for internal consistency. Thereby, a Cronbach’s α > 0.7 was considered acceptable [20]. Furthermore, we tested the impact of item removal on Cronbach’s α.

Concurrent validity was evaluated by using the Pearson’s correlation coefficient (r) between the GR-Scale total score and the lung function parameters forced vital capacity (FVC) and diffusion capacity for carbon monoxide (DLco). The percentage values of the patients’ respective predicted values, based on the GLI database, were used and DLco was corrected for haemoglobin. We categorized the correlations based on their Pearson’s correlation coefficient (r) as follows: <0.3 were classified as weak, those ⩾0.3 to < 0.7 as moderate and those ≥ 0.7 as strong [21].

The known-groups validity was evaluated to analyse the GR-Scale’s ability to distinguish among distinct groups. The GR-Scale total scores between different categories of the following variables were compared: (1) FVC % predicted (> 75%, 75 to 45%, < 45%); (2) DLco % predicted (> 60%, 40 to 60%, < 40%); (3) use of long-term oxygen therapy (LTOT). We further compared the GR-Scale total score between different ILD subtypes (IPF vs. non-IPF). Therefore, we used the independent two-sample t-test and reported the effect size as Cohen’s d.

Descriptive statistics were also calculated for follow-up variables. After the follow-up interviews, we assessed again the concurrent validity by calculating Pearson’s correlation coefficient (r) between the GR-Scale total score and the two lung function parameters and we also compared again the GR-Scale totals between different ILD subtypes (non-IPF vs. IPF). Additionally, the Pearson’s correlation coefficient (r) between the changes in the respective lung function parameters and the changes in the GR-Scale total score between the two surveys were calculated. Furthermore, we divided the patient population into three groups based on their lung function parameter changes ( > + 5%, + 5% to -5%, <-5% FVC% predicted and DLco% predicted) and compared the changes in the GR-Scale total score among these groups.

For statistical analysis SPSS 29, with *p* < 0.05 considered statistically significant, was used and the figures were created with both SPSS 29 and Figma desktop version 116.14.7.

### Ethical approval

This pilot study was approved by the ethics committee of the medical faculty of LMU Munich (project number 22–0651). Written informed consent was obtained from all patients prior to enrolment.

## Results

Between October 2022 and December 2022, the GR-Scale was completed by 80 patients with a multidisciplinary discussion (MDD) based diagnosis of ILD undergoing routine clinical care at our tertiary hospital. All 80 patients filled in the questionnaire without missing information. The patient demographics and baseline characteristics are shown in Table 1. The mean age was 61.74 ± 14.04 years, 44% were females. The mean GR-Scale total score was 18.9 ± 9.03 and the mean ± SD for FVC and DLco were 75.36 ± 22% of predicted and 47.75 ± 18.11% of predicted, respectively.

**Table 1:** Patient demographics and baseline characteristics

Characteristic	Distribution (*n* = 80)
Male: female n	45:35
Age, year	61.74 ± 14.04
Ever smoker n (%)	37 (46.25)
ILD subtype n (%)	
CTD-ILD	32 (40)
cHP	10 (12.5)
IPF	10 (12.5)
NSIP	8 (10)
Sarcoidosis	15 (18.8)
Stage III	9 (60)
Stage IV	6 (40)
uILD	5 (6.3)
Comorbidities	2.86 ± 2.05
LTOT n (%)	24 (30)
GR-Scale total score	18.9 ± 9.03
FVC, %predicted	75.36 ± 22
DLco, % predicted	47.75 ± 18.11

Data are presented as mean ± SD, unless otherwise stated.

Definition of abbreviations: ILD = interstitial lung disease; CTD-ILD = connective tissue disease-associated interstitial lung disease; cHP = chronic hypersensitivity pneumonitis; IPF = idiopathic pulmonary fibrosis; NSIP = nonspecific interstitial pneumonitis; uILD = unclassifiable interstitial lung disease; LTOT = long-term oxygen therapy; FVC = forced vital capacity; DLco = diffusion capacity for carbon monoxide.

### Internal consistency

Cronbach’s α was 0.749 indicating an acceptable internal consistency. Floor and ceiling effects are summarised in Table 2. Two of the five items demonstrated a significant floor effect: “cough” with a floor effect of 21.3% and “depressed mood” with a floor effect of 17.5%. None of the items showed a significant ceiling effect. The analysis of internal consistency by removing individual items showed that excluding any item did not lead to a significant improvement in Cronbach’s α.

**Table 2:** Floor and ceiling effects

Item		Floor (%)	Ceiling (%)
Cough		21.3	1.3
Shortness of breath		2.5	0
Fatigue		7.5	0
Depressed mood		17.5	1.3
Overall sense of wellbeing		1.3	0
Total Score		0	0

### Concurrent and known-groups validity

The GR-Scale total score and the two lung function parameters showed weak but statistically significant inverse correlations, FVC % predicted (*r*=-0.282, *p* = 0.011, 95% confidence interval (CI) [-0.47, -0.07]) and DLco % predicted (*r*=-0.254, *p* = 0.025, 95% CI [-0.45, -0.03]).

**Fig. 1:**
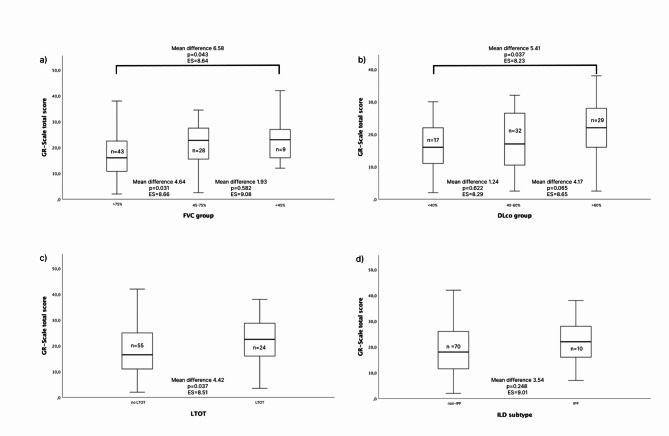
Comparison of GR-Scale total scores between (a) forced vital capacity (FVC) % predicted (> 75%, 45–75%, < 45%); (b) diffusion capacity for carbon monoxide (DLco) % predicted (> 60%, 40–60%, < 40%); (c) long-term oxygen therapy (LTOT) (d) different ILD subtypes (non-IPF vs. IPF). Effect size: ES (reported as Cohen’s d)

To analyse the known-groups validity we compared the GR-Scale total scores between different categories of FVC % predicted and DLco % predicted and use of LTOT (Table 3; Fig. 1). GR-Scale total scores were statistically significantly higher in patients with more severe impairment of FVC % predicted. GR-Scale total scores were also statistically significantly higher in patients with more severe impairment of DLco % predicted. Besides, the GR-Scale total scores were statistically significantly higher in patients receiving LTOT. When comparing the GR-Scale total score between the different ILD subtypes (IPF vs. non-IPF), no statistically significantly difference was found.

**Table 3:** Known-groups validity analysis

Variable	Patients *n*	Mean ± SD GR-Scale total score	Mean difference (95% CI)	*p*-value	ES
FVC					
>75% predicted	43	16.54 ± 8.47	6.58 (0.21–12.94)	0.043	8.64
<45% predicted	9	23.11 ± 9.49			
DLco					
>60% predicted	17	16.27 ± 7.51	5.41 (0.34–10.48)	0.037	8.23
<40% predicted	29	21.67 ± 8.62			
LTOT					
No LTOT	55	17.26 ± 8.3	4.42 (0.28–8.57)	0.037	8.51
LTOT	24	21.69 ± 8.97			
ILD subtype					
non-IPF	70	18.46 ± 9	3.54 (2.52–9.61)	0.248	9.01
IPF	10	22 ± 9.15			

Definition of abbreviations: FVC = forced vital capacity; DLco = diffusion capacity for carbon monoxide; LTOT = long-term oxygen therapy; non-IPF = non-idiopathic pulmonary fibrosis; IPF = idiopathic pulmonary fibrosis; ES: effect size (Cohen’s d).

### Follow-up measurements

Seventy patients (87.5%) completed the GR-Scale questionnaire again after 4.43 ± 1.2months. At follow-up, the mean GR-Scale total score was 18.08 ± 9.92 and the mean values for the lung function parameters were FVC 72.27 ± 22.09% and DLco 48.06 ± 18.61%.

To support the concurrent validity of the baseline, we again calculated Pearson’s correlation coefficients between the GR-Scale total score and the two lung function parameters with the follow-up values. Both lung function parameters showed a moderate inverse correlation, FVC % predicted (*r*=-0.41, *p* < 0.001, 95% CI [-0.59, -0.19]) and DLco % predicted (*r*=-0.445, *p* < 0.001, 95% CI [-0.62, -0.23]).

During follow-up we observed inverse correlations between the changes in the respective lung function parameters and the changes in the GR-Scale total score, FVC % predicted (*r*=-0.376, *p* = 0.001, 95% CI [-0.56, -0.16]) and DLco % predicted (*r*=-0.242, *p* = 0.048, 95% CI [-0.46, 0.002]).

We also compared the GR-Scale total scores again between different ILD subtypes (non-IPF vs. IPF) at follow-up. The mean GR-Scale total score of the non-IPF group was 17.93 ± 9.54 and of the IPF group 19.43 ± 13.72. No statistically significant difference was found between those two groups (*p* = 0.707).

**Fig. 2:**
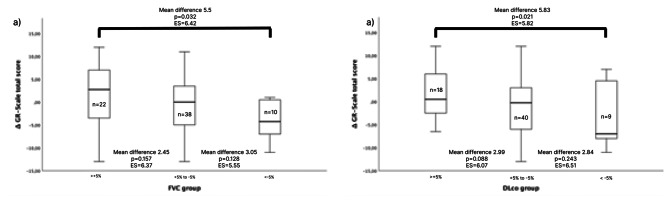
Comparison of the Δ GR-Scale total scores between (a) forced vital capacity (FVC) % predicted ( > + 5%, -5% to + 5%, <-5%) (b) diffusion capacity of carbon monoxide (DLco) % predicted ( > + 5%, -5% to + 5%, <-5%). Effect size: ES (reported as Cohen’s d)

Using a > 5% absolute change of FVC or DLco as cut-off disclosed a statistically significantly difference in GR-Scale total score for both variables (Table 4; Fig. 2).

**Table 4:** Follow-up measurements analysis

Variable	Patients *n*	Mean ± SD ΔGR-Scale total score	Mean difference (95% CI)	*p*-value	ES
FVC					
> +5%	22	1.5 ± 7.20	5.5 (0.5–10.5)	0.032	6.42
< -5%	10	-4 ± 4.06			
DLco					
> +5%	18	1.94 ± 5.19	5.83 (0.94–10.73)	0.021	5.82
< -5%	9	-3.9 ± 6.96			

Definition of abbreviations: FVC = forced vital capacity; DLco = diffusion capacity for carbon monoxide; ES: effect size (Cohen’s d).

## Discussion

In our pilot study we created a German version of the R-Scale [6], the GR-Scale, and evaluated its validity for assessing HRQoL in patients with ILDs. The R-Scale has not yet been translated into another language and this was the first time that the questionnaire was applied and evaluated in a non-English speaking population. Further it has so far only been used in patients with IPF [6], in our study we used the GR-Scale in different ILD subtypes.

The R-Scale, composed of just five items, was created because HRQoL is becoming increasingly relevant in patients with IPF and other ILDs, not only as an endpoint in clinical trials but also in everyday clinical practice, and the existing questionnaires are not used in everyday clinical practice due to their complexity and length [2, 6]. 

The GR-Scale showed an acceptable internal consistency with slight floor/ceiling effects and a good concurrent validity with the two lung function parameters FVC and DLco. Furthermore, the GR-Scale was able to distinguish between patients with different levels of disease severity, differentiating those with mild and severe limitations in the lung function parameters FVC and DLco, and between patients with and without the need of LTOT. Moreover, all 80 patients were able to complete the questionnaire, unsupported by a health care professional, in a complete and evaluable way. Thus, the questionnaire seems to be easy to understand and to apply and could be a simple and quick tool for daily clinic routine to assess the HRQoL of patients with ILD.

For the floor and ceiling effects we have chosen a threshold of 15%, which is often chosen in validation studies [19, 22]. Taking this threshold into account, we were able to detect mild floor effects. We found two significant floor effects for the items “cough” (21.3%) and “depressed mood” (17.5%). No significant ceiling effects were found. In the study by Scallan et al., the item “depressed mood” also showed a significant floor effect of 36%. The item cough did not show a significant floor effect [6]. Cough is one of the leading symptoms of ILDs and has a significant impact on patients’ HRQoL [1, 23, 24]. However, the prevalence of cough varies between different ILD entities. In the work by Cheng et al., cough was less common in patients with systemic sclerosis-associated interstitial lung disease (SSc-ILD; 68%) than in IPF (87%) or cHP (83%) [25]. Similar prevalence of cough has been reported in other studies in ILD [26, 27]. In our analysis, CTD-ILD represent the largest group of our study population with 40%, compared to IPF with 12.5% and HP with 11.3%. This composition of the patient population could have an influence on the floor effect of the item “cough” and might explain why we found a significant floor effect (21.3% of the patients chose the minimum of the item “cough”) and no significant floor effect was found for the item “cough” in the study by Scallan et al. [6], in which only patients with IPF were included.

„Depressed mood” was the second item with a significant floor effect of 17.5%. Depression is frequently observed in patients with ILD, varying between 14 and 49% in different studies and approaches to depression assessment [28]. Depression also has a significant impact on HRQoL in patients with ILD [28–30]. In the study by Scallan et al., the floor effect of the item “depressed mood” of 36% was more than twice as large as in our study (17.5%). The composition of the study population may also be relevant here, which requires additional investigation. A further explanation for the two significant floor effects could be that all patients were already on active therapy for their disease and that they came to the hospital for a routine follow-up examination and not because of complaints or worsening of the disease. Further studies are needed to assess whether floor effects differ between defined study populations.

For concurrent validity, we observed statistically significant correlation between GR-Scale total score and the lung function parameters FVC and DLco. On the one hand, this shows that patients with more severe symptoms also have a more impaired HRQoL. On the other hand, it emphasizes the importance of PROs providing complementary information.

A moderate statistically significant correlation between the total score and lung function (FVC and DLco) was also found in the follow-up measurement. This indicates that the concurrent validity is consistent across repeated measurements. This presence of weak to moderate correlations between the lung functions parameters and the total score supports the validity of the GR-Scale [2, 9].

In addition, our results support the findings of the study by Scallan et al. study, which found moderate correlations between the R-Scale-PF total score and FVC (% predicted), and weak correlations between R-Scale-PF total score and DLco (% predicted) [6].

The GR-Scale was able to distinguish between groups with different disease severities, which were categorized according to the lung function parameters FVC and DLco, and the need for LTOT.

As the disease progresses and symptoms increase, HRQoL becomes more impaired [1–3]. The need for more therapy in this context also has a negative impact on HRQoL. Here, LTOT plays a particularly important role and is a significant limitation in daily life of patients with ILD. LTOT leads to dependence and is a stigma of the disease; thus it leads to impairment of multiple domains of life, including emotional well-being, social participation, and autonomy [1, 2, 4, 31, 32]. This negative impact of LTOT on HRQoL was clearly reflected by the GR-Scale. These results are consistent with the study by Scallan et al. [6].

When comparing the GR-Scale total scores between patients with IPF and non-IPF ILD, no significant differences were found at baseline nor at follow-up, indicating that this tool is applicable to a broad spectrum of ILDs.

The statistically significant correlation between the GR-Scale total score and FVC and DLco at the follow-up measurement indicates that the concurrent validity is consistent across repeated measurements. Moreover, the GR-Scale has shown sensitivity to changes in patients’ health status, even on short follow-up of 4.4 months. The relatively weak correlations also imply that the GR-Scale provides information beyond the lung function parameters.

The GR-Scale was also able to distinguish between patients who improved, declined, or remained stable in the lung function parameters FVC and DLco.

To obtain additional information beyond the conventional measurements of disease severity, the GR-Scale could be used as both a screening tool to identify patients who need further evaluation (as also mentioned in the work by Scallan et al. [6]) and a longitudinal tool to track the patient’s health status over time. When used longitudinally, it could provide a consistent measure of the patient’s HRQoL and thus could make it possible to monitor the progression of the disease and, for example, the effectiveness of treatments. Here, the GR-Scale would indicate how the progression of the disease and treatments affect how the patient “feels and functions” in daily life. This dual functionality increases its utility and usefulness in clinical practice and provides comprehensive insights into the patient’s condition that go beyond what conventional measurements such as lung function parameters can capture.

In our study, the R-Scale was used for the first time in a non-English speaking population, the GR-Scale allows the assessment also in German speaking populations, thus extending the applicability. It is also the first time that the questionnaire has been used not only in IPF but also in other ILD subtypes. By including HRQoL measurements in clinical practice, we gain valuable insights into patients’ health status that go beyond traditional measures like lung function parameters. This understanding is essential for both IPF and other ILD patients, as their quality of life can also be affected [3–5, 7]. We also found no significant difference in the comparison of GR-Scale total scores between IPF and non-IPF ILDs. Our study is a first step towards the applicability of the GR-Scale in non-IPF ILDs. This extension demonstrates the versatility of the questionnaire and its potential suitability for assessing quality of life in different types of ILD.

There are some limitations to our study. First, the study population was relatively small, heterogenous, enrolled in a single centre, and the interval between the two interviews varied among patients, ranging from 3 to 6 months. Second, we used only the two lung function parameters FVC and DLco, as physiological measures, as these were collected in all patients during routine care. However, it would also be of interest to analyse the relationship between GR-Scale and exercise test such as the 6 min walk test or disease extent on High-resolution computed tomography (HRCT). Furthermore, in this study we did not compare the GR-Scale with other questionnaires that can determine HRQoL in patients with ILD and which have already been validated. While the validity of the R-Scale in IPF was moderate to high compared to established questionnaires like K-BILD and EQ-5D-5L by Scallan et al. [6], this important aspect needs to be considered for non-IPF ILDs in future studies.

## Conclusion

HRQoL plays a crucial role in patients with ILDs, and it is important to measure it not only in studies but also in daily clinical practice in order not to miss important information about the patients’ health status. For this purpose, the GR-Scale is a simple and quick tool to measure HRQoL in patients with ILDs. The GR-Scale showed acceptable internal consistency, good concurrent validity over repeated measurement and a good known-groups validity. It was also sensitive to changes in patients’ health status over time. In conclusion, our study provides preliminary evidence that the GR-Scale is clinically useful by being anchored in lung physiology but providing additional compact clinical information which authentically reflects how patients feel and function in daily life.

## Electronic supplementary material

Below is the link to the electronic supplementary material.

Supplementary Material 1. Additional files: The GR-Scale (German version of the R-Scale) is provided in the additional file 1 (.pdf).

## Data Availability

The data will be made available on request and with consideration of further evaluations of our study team (contact: Direktion.Med5@med.uni-muenchen.de).

## Abbreviations

cHP: Chronic hypersensitivity pneumonitis
CI: Confidence interval
CTD-ILD: Connective tissue diseases-related ILD
DLco: Diffusion capacity for carbon monoxide
EQ-5D-5L: EuroQol Five-Dimensional Five-Level questionnaire
FVC: Forced vital capacity
GR-Scale: German version of the R-Scale
HRCT: High-resolution computed tomography
HRQoL: Health related quality of life
ILDs: Interstitial lung diseases
IPF: Idiopathic pulmonary fibrosis
K-BILD: King’s Brief Interstitial Lung Disease questionnaire
LTOT: Long-term oxygen therapy
MDD: Multidisciplinary discussion
NSIP: Non-specific interstitial pneumonia
PROs: Patient reported outcome measures
R-Scale-PF: Raghu scale for pulmonary fibrosis
SSc-ILD: Sclerosis-associated interstitial lung disease
uILD: Unclassifiable interstitial lung disease
WHO: World Health Organization
