# Supplementary material for: Assessing health-related quality of life in patients with interstitial lung diseases
Source: BMC Pulm Med. 2024 Sep 13;24:452. doi: 10.1186/s12890-024-03262-9 (PMC11401309; doi:10.1186/s12890-024-03262-9)
Supplement: Supplementary file 1 — Supplementary Material 1. Additional files: The GR-Scale (German version of the R-Scale) is provided in the additional file 1 (.pdf). [file 12890_2024_3262_MOESM1_ESM.pdf]

Visuelle Analogskalen zur Erfassung des Gesundheitszustands

Dieser Fragebogen dient dazu, die Auswirkungen Ihrer Lungenerkrankung auf Ihren Gesundheitszustand zu beurteilen. Bitte wählen Sie auf jeder Skala einen Wert aus, je nachdem, wie Sie sich in den <u>letzten 2 Wochen</u> gefühlt haben.

kein — stark

0 1 2 3 4 5 6 7 8 9 10

Husten

keine — stark

0 1 2 3 4 5 6 7 8 9 10

Kurzatmigkeit

keine — stark

0 1 2 3 4 5 6 7 8 9 10

Abgeschlagenheit

keine — sehr

0 1 2 3 4 5 6 7 8 9 10

Gedrückte Stimmung (Depression)

Sehr gut — Sehr schlecht

0 1 2 3 4 5 6 7 8 9 10

Allgemeines Wohlbefinden
